# How Woman‐Centred Care Is Experienced and Understood in Maternity Services by Women and Professionals: A Rapid Review

**DOI:** 10.1111/scs.70086

**Published:** 2025-07-10

**Authors:** Soma Gregory, Louise Caffrey, Deirdre Daly, Greg Sheaf

**Affiliations:** ^1^ School of Social Work and Social Policy Trinity College Dublin Dublin Ireland; ^2^ School of Nursing and Midwifery Trinity Centre for Maternity Care Research (TCMCR), Trinity College Dublin Dublin Ireland; ^3^ The Library of Trinity College Dublin Dublin Ireland

**Keywords:** maternity care policy, maternity services, maternity services high‐income countries, midwifery, person‐centred care, rapid review, woman‐centred care

## Abstract

**Background:**

Woman/person‐centred care is a key policy objective for maternity services in many high‐income countries, both strategically and in terms of service delivery. This approach to care prioritises the individual woman's needs and aspirations over those of professionals or institutions. However, the concept remains ambiguous and practical implementation guidelines are lacking, leading to concerns that it is being practised tokenistically.

**Aim:**

To explore how woman‐centred care in maternity services in high‐income countries is experienced and understood by women and professionals.

**Methods:**

A rapid review was conducted to synthesise evidence on woman‐centred care from women's and clinicians' perspectives. Five bibliographic databases, CINAHL, Maternity and Infant Care, MEDLINE, PsycINFO and Web of Science, were searched systematically in December 2023. Citations eligible for inclusion were peer‐reviewed quantitative, qualitative and mixed‐methods empirical studies conducted in high‐income countries and literature reviews reporting on studies conducted in high‐income countries. Title and abstract screening, full‐text screening, data extraction and quality appraisal were performed, with 10% of records double‐screened to ensure consistency and minimise bias. Methodological quality was assessed using the Mixed Methods Appraisal Tool. Extracted data were synthesised thematically.

**Findings:**

In total, 5295 records were retrieved; after deduplication, 2707 records were screened, and 24 studies met the inclusion criteria. The review identified inconsistencies in how woman‐centred care was experienced and understood at all levels of service provision, from policy directives to institutional practices. Diverse understandings of woman‐centred care among stakeholders were found to influence interactions. Several studies revealed that professionals' varying interpretations affected their attitudes and approaches to care, while some women were found to hold differing expectations about woman‐centred care provision.

**Conclusion:**

To realise the policy intention of woman‐centred care, further context‐specific investigations are needed to provide evidence and evaluation to inform implementation strategies.

## 1 Introduction

The United States Institute of Medicine's (2001) seminal report ‘Crossing the Quality Chasm’ identified person‐centred care as essential to healthcare quality and patient safety [1], including within maternity services. The concept of care‐provision being centred on the individual who is receiving care has a long, multidisciplinary history and comprises several variations in terms [2, 3], including patient‐centred, client‐centred, family‐centred, person‐centred and woman‐centred care, among others. Reframing patient‐centred as person‐centred care in the early 2000s [2] has been described as anti‐reductionist, emphasising that patients are persons, not merely their conditions [2, 3]. Defining person‐centred care is complex as it encompasses multiple meanings and is widely regarded as a multidimensional concept [3]. Nonetheless, person‐centred care is generally characterised by respect and responsiveness to an individual's preferences, needs and values, with these values guiding all clinical decisions [1]. Although conceptual frameworks exist, practical guidance remains limited [4].

Woman‐centred care (WCC) derives from person‐centred care (PCC), and while often used interchangeably, WCC typically refers to a partnership between the woman and her midwife or maternity healthcare professional [5]. Both concepts, however, have been criticised as vague in practice [6, 7], with stakeholders—women, midwives and doctors—purported to hold differing views on their meaning and implementation [4, 8]. Concerns have been raised internationally about the tokenistic application of these concepts [4, 7, 9]. Therefore, given these different philosophical approaches to care, it is essential to explore and clarify both professionals' and service‐users' understandings and experiences of WCC within maternity services. Additionally, care should not be seen as something ‘delivered’ to a woman, but as co‐constructed through interactions among stakeholders throughout a woman's childbirth journey [10, 11]. Reflecting this, the World Health Organization (WHO) advocates for collaborative, informed decision‐making that enables the woman to manage her own care [12].

As noted, the terms WCC and PCC are often used interchangeably, which can be contentious [9, 13]. However, since it remains unclear which version of *‐centred‐care maternity services professionals align with, it seems prudent to retain both terms until clarity is established.

### 1.1 Woman‐Centred Care in Maternity Services

WCC is synonymous with midwifery and is reflected in the International Confederation of Midwives' statement on the Philosophy of Midwifery Care [14]. WCC care has become a key policy objective for maternity services internationally, both strategically and operationally [15, 16, 17]. Despite its widespread endorsement, contextual considerations provide a strong rationale for investigating the implementation of WCC in individual countries, or clusters of countries, with relatively comparable maternity services [18]. As maternity services provision and access to care differ considerably between High‐, Middle‐ and Low‐income countries, this rapid review focuses on findings from studies in High‐income Countries (HICs) with Organization for Economic Co‐operation and Development (OECD) membership in 2024.

#### 1.1.1 Defining Woman‐Centred in Pregnancy and Childbirth

Leap states that midwife‐provided WCC should prioritise the individual woman's needs, aspirations and expectations above institutional or professional interests, emphasising choice, control and continuity of care from known caregivers. The woman's needs (social, emotional, physical, spiritual and cultural needs) and her family's needs are defined and negotiated by the woman *herself*, recognising the woman's expertise in decision‐making [5]. However, this approach overlooks the role of other professionals involved in maternity care, which is important as integrated care models are increasingly promoted for delivering safe, effective and high‐quality services [19].

Recently, there has been a growing body of research on defining and measuring WCC globally [7, 8, 9, 20]. Notably, Brady and colleagues' evidence‐based definition excludes continuity of care or carer as a core attribute of WCC, which is central to Leap's definition [5], because midwives participating in their international Delphi study did not identify it as essential [8]. (For an in‐depth comparison of several definitions [5, 9, 21], please see Supplementary Information File 1). However, midwives in Brady et al.'s Delphi study practised in High‐, Middle‐ and Low‐Income countries with diverse maternity care systems [8]. Therefore, this finding may not hold in countries with relatively comparable maternity services.

### 1.2 Divergent Approaches to Birth

The aim of WCC was to increase the likelihood that a woman will achieve a positive birthing experience; defined as one which ‘fulfil[s] or exceed[s] prior personal and socio‐cultural beliefs and expectations’ (p. 1) [22]. The literature highlights divergent values and ideologies surrounding pregnancy and birth [23, 24, 25, 26], which are biological events embedded in social and cultural contexts [27], and thus socially constructed [10]. Consequently, childbirth holds different meanings depending on individual circumstances and values. Indeed, Van Teijlingen [25] positions birth approaches on a continuum from a medicalised approach at one end to a de‐medicalised/social approach at the other.

The medical model views childbirth as inherently risky requiring hospital‐based care and obstetric oversight, with safety only confirmed in retrospect [23, 24, 25]. In contrast, the social model views childbirth as a physiological process which, for most women, proceeds without complications or requiring medical intervention [23, 26]. This social model prioritises a woman's sense of well‐being and childbirth experience as key outcomes [23]. Notwithstanding these seemingly opposing approaches to childbirth, it has been concluded that elements of both approaches are required to provide quality and ‘fit for purpose’ maternity care [23] Therefore, it is essential that care is individualised and responsive to each woman's needs, especially where medicalised care is the only available option.

## 2 Methods

### 2.1 Review Aim

The aim was to synthesise evidence on the concept of WCC as it relates to the continuum of childbirth and was guided by the research question:

How is WCC experienced and understood by women as service‐users and professionals (midwives and obstetricians) who provide care in maternity services in high‐income countries (HICs)?

### 2.2 Design and Protocol

A rapid review was selected as the most suitable approach as it is a robust and rigorous way to expedite and streamline the traditional approach for systematic reviews [28, 29]. The review was guided by protocols for conducting rapid reviews [29, 30], and the Preferred Reporting Items for Systematic Reviews and Meta‐Analysis 2020 (PRISMA) Protocols checklist was utilised [31].

### 2.3 Eligibility Criteria

Peer‐reviewed empirical studies conducted in HICs, and literature reviews focused on studies conducted in HICs, published in English were included. Discussion papers and commentaries were excluded. The inclusion and exclusion criteria applied in this rapid review are presented in Table 1 below.

**TABLE 1:** Inclusion and exclusion criteria.

Inclusion criteria	Exclusion criteria
English language only. Peer reviewed. Primary research. Literature reviews. High‐income countries with OECD membership in 2024 with similar healthcare systems. The aim/objective/purpose of the study is explicitly on woman (person/etc.)‐centred care in the context of maternity services. WCC delivered by different professionals (midwives, obstetric nurses and doctors), in different settings (home, hospital and birth centres).	The aim/objective of the study does not explicitly investigate woman (person/etc.)‐centred care in the context of maternity services. Middle‐ and low‐income countries. Articles on how to do woman‐centred research (e.g., methodological approaches) or the development/validation of scales for measurement of WCC or which develop models of care. WCC delivered in other settings. Other reproductive health care: For example, contraception, abortion care or gynaecological care, or health promotion advice, for example, nutrition, smoking, alcohol, etc. Literature which explores the education of midwives, obstetricians, GPs or other healthcare professionals about WCC. Literature about how to deliver specific interventions, clinical techniques, programmes or a drug (e.g., nitrous oxide) Non‐empirical papers—for example, theory only. Abstracts and conference papers.

### 2.4 Information Sources and Search Strategy

A systematic search strategy suitable for rapid reviews [32] was constructed using keywords and controlled vocabulary, drawing on prior studies on WCC and the expertise of the author team, including an Information professional. The search used ‘person’, ‘woman’ and ‘women’ as the prefix to ‘centred’. ‘Patient’ was also included if it was included as part of MeSH headings. Studies using alternative terms like ‘client’ were included if the focus remained on the service‐user in *‐centred care. This search was piloted in Web of Science—and then adapted for CINAHL, Maternity and Infant Care, Medline and PsycInfo from inception to 13 December 2023. The MEDLINE search example is shown in Table 2.

**TABLE 2:** Example of database search.

Example search strategy in MEDLINE (Ebsco)
MH ‘Patient‐Centered Care+’ OR TI((woman OR women OR person) N2 (centre* OR center*)) OR AB((woman OR women OR person) N2 (centre* OR center*)) AND MH ‘Parturition+’ OR MH ‘Labor, Obstetric+’ OR TI(Childbirth* OR birth* OR pregn* OR labour OR labor OR intrapartum OR postpartum OR matern* OR homebirth* OR midwif* OR obstetric*) OR AB(Childbirth* OR birth* OR pregn* OR labour OR labor OR intrapartum OR postpartum OR matern* OR homebirth* OR midwif* OR obstetric*) AND MH ‘Qualitative Research+’ OR TI(experienc* OR view* OR perception* OR perceiv* OR perspective* OR attitude* OR belief* OR narrat* OR qualitative OR concept* OR model* OR understand* OR understood OR empirical) OR AB(experienc* OR view* OR perception* OR perceiv* OR perspective* OR attitude* OR belief* OR narrat* OR qualitative OR concept* OR model* OR understand* OR understood OR empirical)

Reference lists of included studies were screened for additional relevant papers, and forward citation chaining was conducted to identify studies citing those included.

### 2.5 Record Screening and Study Selection

In December 2023, all records retrieved from the five bibliographic database searches were imported into Covidence [33,34]. A pilot screening was conducted to ensure consistency among the three reviewers. At each stage, 10% of records were double‐screened independently by the second and third authors (5% each) [28], with any conflicts agreed by consensus. This approach aimed to minimise bias and uphold methodological rigor.

### 2.6 Data Extraction, Synthesis and Quality Assessment

A tailored data extraction form was designed in Covidence [33, 34]. The first author extracted data from all included studies, with 10% double‐extracted by a second reviewer, as described above. Discrepancies were resolved through discussion and consensus. Extracted data were exported to Microsoft Excel and analysed using Braun and Clarke's six‐phase thematic analysis [35, 36]. A coding framework was developed to identify themes related to how WCC is experienced and understood by women and professionals. Methodological quality was assessed using the Mixed Methods Appraisal Tool (MMAT), when appropriate [37] with 10% of studies double‐screened by a second reviewer.

## 3 Results

### 3.1 Results of the Selection Process

**FIGURE 1:**
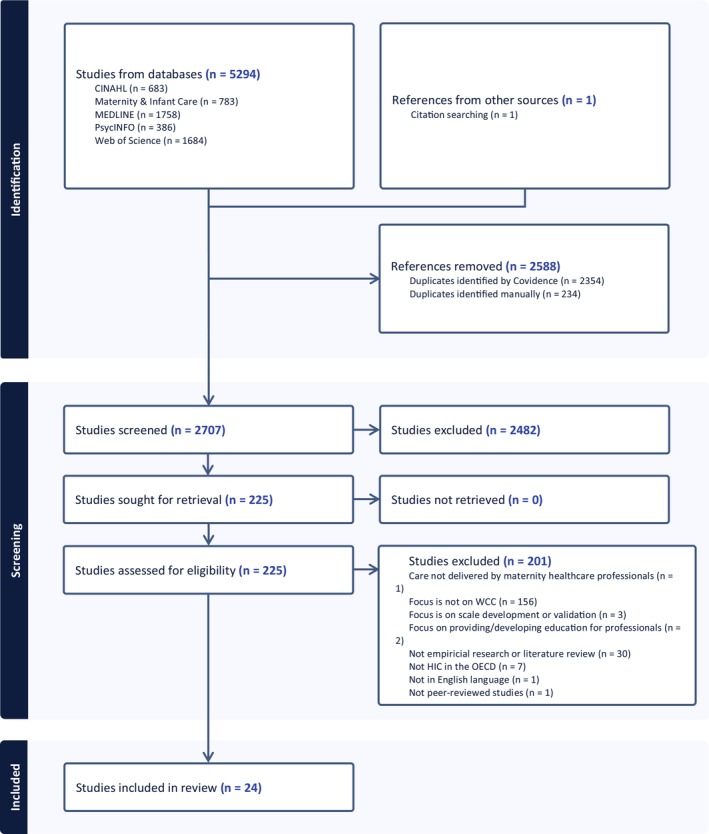
PRISMA 2020 flow diagram.

A total of 5295 records were retrieved. After deduplication (*n* = 2588), 2707 records were screened by title and abstract, 225 reports were screened at full text, and 24 records met the inclusion criteria. Figure 1 presents the PRISMA 2020 diagram.

### 3.2 Characteristics and Quality Appraisal of Included Studies

Twenty of the 24 included studies were primary empirical studies [38, 39, 40, 41, 42, 43, 44, 45, 46, 47, 48, 49, 50, 51, 52, 53, 54, 55, 56, 57] and four were secondary data analyses. These studies were conducted in Australia (*n* = 2), Belgium (*n* = 1), Germany (*n* = 1), Iceland (*n* = 1), Ireland (*n* = 1), Japan (*n* = 1), Netherlands (*n* = 6), Sweden (*n* = 4), Switzerland (*n* = 1), United Kingdom (*n* = 3) and United States (*n* = 4) between 1998 and 2000, and published between 2001 and 2023. Four papers did not report data collection dates [42, 45, 47, 56].

Ten studies employed qualitative methods [38, 39, 42, 44, 47, 48, 52, 53, 55, 57], six used quantitative methods [40, 41, 46, 49, 50, 51] and four used mixed‐methods [43, 44, 54, 56]. The four secondary data analyses included a concept analysis [21], critical appraisal and synthesis [58], comparative policy review [59] and integrative review [60]. Nine studies focused on women and birthing people only [40, 41, 42, 46, 49, 50, 51, 52, 55], six involved midwives only [39, 44, 45, 47, 56, 57], one included women and midwives [54] and three involved various maternity healthcare professionals [38, 43, 53]. Only one involved all stakeholders, for example, service users and professionals [48].

Overall, the six quantitative studies involved 6310 women and birthing people, 490 healthcare professionals and 771 midwives, and the 10 qualitative studies included 164 women, 225 midwives and 94 other healthcare professionals.

Nineteen papers were appraised using the MMAT; 17 papers received a rating of 5 and two scored 4. The MMAT was not applicable to five papers [21, 52, 58, 59, 60], four did not collect primary data [21, 58, 59, 60] and one was a rapid review with qualitative PPI feedback [52] which did not meet the tool's parameters. Full details of the studies reviewed are provided in Table 3.

**TABLE 3:** Data extraction table.

Author (year)	Study aim/objective	Year of study	Country	Type of research	Methodology	Data collection method	Type of secondary research study	Sample	Findings on understanding	Findings on experiences	MMAT^a^ Rating
Adams et al. (2018)	To explore how clinicians describe patient‐centred care and how the concept is understood in their practice. It sought to investigate (i) How do clinicians describe patient‐centred care? (ii) How does being patient‐centred affect how care is delivered? (iii) Is this different for vulnerable populations? And if so, how?	2015	United States	Primary Research	Qualitative	Interview; Focus Group	—	Purposively sampled Professionals; Obstetricians (*n* = 10) Family practitioners (*n* = 1) Midwives (*n* = 4) Physician's assistants (*n* = 1)	X	X	*****
Ahlund et al. (2018)	The aim in this study was to investigate how midwives experienced implementing woman‐centred care during second stage of labour.	2013	Sweden	Primary Research	Qualitative	Interview; Focus Group	—	Professionals Midwives (*n* = 20) working in two different labour wards in Sweden 9 had < 5 years experience, 11 had > 5 years experience	X	X	*****
Altman et al. (2022)	The purpose of this paper is to describe findings from a study that used two validated scales to examine factors associated with experiences of person‐centred care during pregnancy and birth among Black birthing people in California.	2020	United States	Primary Research	Quantitative	Questionnaire	—	Service‐users (*n* = 234) Purposive sample of women and birthing people who identified as Black or African American	—	X	****
Attanasio et al. (2022)	Person‐centred care has been increasingly recognised as an important aspect of health care quality, including in maternity care. Little is known about correlates and outcomes of person‐centred care in maternity care in the United States.	2009–2011	United States	Primary Research	Quantitative	Questionnaire	—	Service‐users (*n* = 2892) Purposive sample of individuals who gave birth to a first baby in a Pennsylvania hospital.	—	X	*****
Bergman & Connaughton (2013)	This qualitative study privileges patients' voices and adds a cultural dimension to existing health communication research on PCC through an empirical investigation of 48 Hispanic prenatal care patients' understandings and expectations of PCC	Not stated	United States	Primary Research	Qualitative	Interview	—	Service‐users (*n* = 48) Hispanic prenatal care patients (Mexico, *n* = 36; Honduras, *n* = 4; El Salvador, *n* = 3); Costa Rica, (*n* = 1); Argentina, (*n* = 1); Guatemala, *n* = 1; Nicaragua, *n* = 1; and US (*n* = 1)	X	X	*****
Dahlen et al. (2023)	The aims of this paper were to examine reasons behind reported dissatisfaction, and compare the WCC Strategy against similar international strategies/plans. The four guiding values in the WCC strategy: safety, respect, choice and access were used to facilitate comparisons and provide recommendations to governments/health services enacting the plan.	N/A	Australia; Canada; Ireland; New Zealand; United Kingdom	Secondary Research		Other	Comparative review of policy and strategy documents	Maternity strategies from comparable HIC (*n* = 9 policy documents in total)	X	—	N/A
de Labrusse et al. (2016)	We sought to identify and critically appraise the literature to identify which definition of PCC is most relevant for maternity services.	N/A		Secondary Research			Critical appraisal and synthesis of the literature	Definitions for PCC were critically appraised (*n* = 6)	X	—	N/A
Floris et al. (2023)	[…] this study aimed to explore HCPs definitions of WCC and identify the degree of agreement and knowledge regarding perinatal indicators when a WCC model of care is implemented.	2019	Switzerland	Primary Research	Mixed‐methods	Interview; Questionnaire	—	Professionals' Cross sectional questionnaire (*N* = 318): Midwives and nurses (*n* = 256) Medical doctors (*n* = 62) Purposively sampled professionals' interviews (*N* = 15): Midwives (*n* = 7) Midwives/Nurse Team Manager or Advanced practitioners (*n* = 2) Jnr Obstetricians (*n* = 2) Snr obstetricians (*n* = 2) Paediatrician (*n* = 1) Anaesthesiologist (*n* = 1)	X	X	*****
Fontein‐Kuipers et al. (2019)	To reveal midwives' distinct perspectives about woman‐centred care, exploring how these perspectives shape midwifery practice.	Not stated	Netherlands	Primary Research	Mixed‐methods	Interview; Q‐methodology	—	Professionals Purposive sample of Dutch community‐based midwives (*n* = 48)	X	X	*****
Fontein‐Kuipers et al. (2019)	To examine the woman‐centredness of maternity care providers from the woman's perspective	2016–2018	Netherlands	Primary Research	Quantitative	Questionnaire	—	Service‐users Pregnant/antenatal women (*n* = 131) Postpartum women from antenatal sample (*n* = 100)	—	X	****
Fontein‐Kuipers et al. (2018)	We aim to provide a clear conceptual foundation of woman‐centred care for midwifery science and practice	N/A	Australia; New Zealand; Sweden; United Kingdom	Secondary Research			Concept Analysis	Research studies (*n* = 8) (note from HIC OECD countries)	X	—	
Fontein‐Kuipers et al. (2016)	The purpose of this study was to obtain self‐referent perspectives of influencing factors of woman‐centred care of Dutch midwives.	2015	Netherlands	Primary Research	Qualitative	Interview	—	Purposively sampled professionals; Midwives (*n* = 10)	X	X	*****
Hunter et al. (2017)	The aim of this study was to explore the concept of WCC during pregnancy and birth within the Irish context, and, through women's and clinicians' views, experiences and perspectives, identify the key elements of WCC so that it might be better understood.	2015	Ireland	Primary Research	Qualitative	Interview; Focus Group	—	Service‐users and professionals (*n* = 31); Women (postnatal) (*n* = 11) Midwives (*n* = 10) Obstetricians (*n* = 5) General practitioners (*n* = 5)	X	X	*****
Iida et al. (2012)	The objectives of this study were to ask (a) what are the perceptions and comparison of WCC at Japanese birth centres, clinics and hospitals and (b) what are the relationships between WCC and three dimensions of women's birth experience: (1) satisfaction with care they received during pregnancy and birth, (2) sense of control during labour and birth and (3) attachment to their new born babies	2008	Japan	Primary Research	Quantitative	Questionnaire	—	Service‐users (*n* = 482) Convenience sample of women in Japan who had a singleton pregnancy in selected birth facilities	—	X	*****
Johnson et al. (2003)	The aim of this study is to compare various aspects of women‐centred care (continuity, information, choice and outcomes) and satisfaction in low risk women receiving maternity services from midwives using a partnership caseload approach, with women receiving standard hospital care.	1998–2000	Australia	Primary Research	Quantitative	Questionnaire	—	Service‐users; purposively sampled women comprising antenatal (*n* = 637) and postnatal (*n* = 571)	—	X	*****
(Fontein) Kuipers et al. (2021)	To examine pregnant women's perceptions of the interpersonal action component of woman‐centred care by primary care midwives working in different sized practices.	2017–2018	Netherlands	Primary Research	Quantitative	Questionnaire	—	Service‐users Convenience sample of women who received midwifery‐led primary care (*n* = 553)	—	X	*****
Leinweber et al. (2022)	To formulate an inclusive woman‐centred definition of a positive childbirth experience	2021	Belgium; Germany; Iceland; Netherlands; Sweden; United Kingdom	Primary Research	Qualitative	Other: Professionals: Interdisciplinary expert input, Feedback from clinicians and researchers Service‐users: PPI input from women (*n* = 42) in 6 countries via service‐user groups and social media fora.	—	Service‐users (*n* = 42) PPI input from women (*n* = 42) in six countries (Belgium, Germany, Iceland, The Netherlands, Sweden, and the United Kingdom)	X	X	N/A
Lundgren et al. (2019)	To explore whether, when adopted by midwives on labour wards, a midwifery model of woman‐centred care (MiMo) was useful in practice from the viewpoint of a variety of health professionals	2015–2016	Sweden	Primary Research	Qualitative	Focus Group	—	Professionals Sampled from the intervention sites; Midwives (*n* = 16) Managers (*n* = 8) Obstetricians (*n* = 8) Assistant nurses (*n* = 11)	X	X	*****
Lundgren et al. (2022)	The aim was to explore and analyse a midwifery model of woman‐centred care (MiMo) in relation to the use of oxytocin for augmentation of labour and to women's childbirth experiences.	2014–2017	Sweden	Primary Research	Mixed‐methods	Focus Group; Questionnaire; Other: Service‐users' hospital data	—	Professionals; Midwives (*n* = 11) Service‐users; Women's hospital data (*n* = 6882) Service‐user questionnaires (*n* = 810)	—	X	*****
(Hollins) Martin & Bull (2009)	Allegiance to a hierarchical system driven by protocols and orders from the top‐down, at the same time as providing ‘woman‐centred’ care is often unattainable. In order for a midwife to action the woman's choice, resourceful thinking may be required. This paper aims to examine this issue.	Not stated	United Kingdom	Primary Research	Qualitative	Interview	—	Professionals; Convenience sample of Midwives (*n* = 20)	—	X	*****
Petit‐Steeghs et al. (2019)	[…] the aim of this study is to analyse the perspectives of women on maternity care and to provide recommendations on how to achieve client‐centred care.	2014–2016	Netherlands	Primary Research	Qualitative	Interview; Focus Group	—	Service‐users Purposive sample of women who gave birth less than 1 year ago in the Netherlands (*n* = 63)	X	X	*****
Pope et al. (2001)	This paper presents findings related to the provision of woman‐centred care from a national research and development study.	Not stated	United Kingdom	Primary Research	Mixed‐methods	Interview; Focus Group; Questionnaire; Other: Case study sites (*n* = 3)	—	Professionals; Simple random sample technique Questionnaire: Midwives (*n* = 771) Co‐ordinating supervisors of midwives (*n* = 172) Focus group/Interviews Midwives (*n* = 90)	X	X	*****
Severinsson et al. (2017)	The aim was to evaluate the current state of knowledge pertaining to patient safety and its link to person‐centred care.	N/A	Australia; Ireland; Japan; Netherlands; United States	Secondary Research			Integrative review	Studies (*n* = 12)	X	X	N/A
Stulz et al. (2022)	To explore midwives' experiences about how COVID‐19 impacted their ability to provide woman centred care	2020	Australia	Primary Research	Qualitative	Interview	—	Professionals; Purposive sample of midwives from all models of care (*n* = 26)	—	X	*****

^a^ The Mixed Methods Appraisal Tool (MMAT) is a validated instrument used to critically assess the methodological quality of qualitative, quantitative and mixed methods studies included in systematic reviews [37].

## 4 Findings

### 4.1 Women's Understanding and Experiences of WCC

Four studies published between 2013 and 2022 presented findings relating to women's understanding of woman/person‐centred care (W/PCC) [42, 48, 52, 55]. These universally reported that women, as maternity services users, hold disparate understandings about what constitutes W/PCC [42, 48, 52, 55]. Some studies indicated that certain woman/service‐user characteristics could also influence how care is experienced [40, 41, 42, 55]. A 2015 study which included 10 women maternity service users in Ireland [48] found that participants emphasised the importance of normalising pregnancy, including minimising interventions and respecting women's wishes and individual experiences as part of WCC [48]. However, they also equated medicalised care with safety and normality [48].

A 2021 European study developed a woman‐informed definition linking WCC to a positive childbirth experience, which included feeling ‘*supported, in control, safe, and respected’* (p. 364) [52]. This definition was developed through interdisciplinary expert input and refined with feedback from 42 women across six European countries, reinforcing the centrality of control and respect; core elements of WCC in the literature [5, 9]. A Dutch study with 63 women found that they wanted to be treated as a *whole* person who was interacting with another person to arrive at mutually appropriate decisions based on clear information from within a flexible and competent maternity system [55]. Both of these studies [52, 55] stressed the importance of the interactions between the woman and the system, particularly in relation to respect. While Leinweber et al. emphasised a woman's control as essential [52], Petit‐Steeghs focused on reaching ‘mutually‐appropriate decisions’ [55], suggesting a more collaborative model involving professional input, rather than the woman's interpretation of her needs being of most importance.

Offering a different perspective, a US study with 48 migrant Hispanic women found that perceptions about ‘patient‐centred care’ diverged from standard definitions [42]. Participants prioritised friendly interpersonal behaviours, effective medical attention, language proficiency, understanding of information and the elimination of racism. Notably, involvement in decision‐making or control were not mentioned. Similarly, in the Dutch study, women with lower educational attainment valued warm and empathetic interactions with maternity care professionals [55]. These findings highlight how cultural and socio‐demographic factors shape expectations of care and WCC, sometimes differing from dominant themes centred on autonomy and control.

Two US studies used validated scales to examine factors influencing PCC experiences [40, 41]. One focused on 234 black women and birthing people [40], while the other surveyed 2892 first‐time mothers at 1 and 6 months postpartum [41]. Lower PCC scores were associated with socio‐economic disadvantage [40, 41], lower educational attainment [40, 41], type of birth experienced and/or maternal birth complications [41] and lack of continuity of care [40]. Higher scores were linked to good/excellent self‐reported health in pregnancy [40], positive attitudes to vaginal birth [41], racial concordance with maternity professionals and continuity of care [40]. Additionally, higher PCC scores among first‐time mothers correlated with improved mental and physical health postpartum [41], highlighting the positive impact of PCC on maternal outcomes.

All these studies which explored women's understandings and experiences of W/PCC illustrate that there are wide variations in what women perceive W/PCC to be, which are highly individual and context‐specific.

### 4.2 Professionals' Understanding and Experiences of Providing WCC

The included studies reveal varied understandings and awareness of W/PCC among healthcare professionals and within maternity services [21, 38, 43, 44, 45, 48, 58]. Two studies explicitly identified potential misunderstandings, or lack of consensus, in relation to the provision of WCC [38, 48]. A 2015 Irish study found inconsistencies in interpretations among stakeholders and concluded that WCC, as understood by participants, was not commonly practised [48]. Similarly, a Dutch study reported that despite its ethical foundations, WCC was not standard practice and required significant shifts in attitudes and beliefs for implementation [21]. In the US, maternity healthcare professionals highlighted the importance of trust in ‘patient'‐centred care, an element which the authors note is absent from the Institute of Medicine's 2001 definition [38].

Two Dutch studies with midwives revealed divergent perspectives on WCC within the profession [44, 45]. A 2016 qualitative study with 10 midwives found that while WCC was generally seen as important and positive, interpretations varied from being the *‘essence of midwifery,’ to* a *‘complicated’* concept or a ‘*temporary trend*’—the latter of which questions the sincerity of intention to provide WCC (p. 23) [44]. The second study, using a Q‐methodology and interviews with 48 Dutch community midwives, identified four distinct factors/perspectives about WCC [45], with differing emphasis on the woman's autonomy, quality standards and self‐management [45]. The authors found that midwives' life‐experiences influenced how they interpreted and practised WCC [44]. These findings suggest that midwives' personal experiences and beliefs significantly shape how WCC is understood and practised across settings.

Lastly, studies across countries revealed varying levels of awareness among maternity healthcare professionals regarding the effects and benefits of W/PCC [38, 43, 44], as well as divergent approaches to care provision [53]. In Switzerland, professionals emphasised a woman's personal resources, history, and environment when defining WCC (p 15) [43], but omitted elements like control, which are central to Leap's definition [5]. A 2015–2016 Swedish midwifery model of WCC intervention study further highlighted disparities in perceptions of risk, complications and leadership roles during childbirth, which influenced the birthing environment and decision‐making processes [53]. These differences may affect interactions and decision‐making, significantly shaping the birth experience for the woman and all involved [53].

### 4.3 Varied understandings of the domains of WCC

#### 4.3.1 A Woman's Self‐Authority in Relation to Choice and Control

A prominent theme emerging from the studies reviewed related to women's and clinicians' understanding of a woman's self‐authority as a component of WCC [38, 43, 44, 45, 47, 48, 49, 52, 56]. This involved exercising choice, control and a woman's (cap)ability to lead or participate in care decisions. W/PCC stipulates that the woman is involved in all care decisions during pregnancy and birth, including the valid choice to defer decision‐making to a maternity care professional. Two studies specifically identified control as essential to a positive birth experience for women [49, 52].

While professionals often endorsed choice and informed decision‐making as central to W/PCC [38, 48, 56], several studies revealed real or perceived limitations in practice [38, 43, 44, 45]. In a Dutch study, most midwives viewed women leading care decisions as impractical, citing unrealistic or unsafe expectations [44]. In a UK study, midwives perceived women from lower socio‐economic backgrounds as less interested in being fully informed [56], while US professionals noted challenges in shared decision‐making when a woman had unmet non‐medical needs [38]. In a Swiss study, the woman's need to have control over her childbirth experience and her expertise in this regard were largely absent from professionals' definitions [43].

#### 4.3.2 Interpersonal Relationships, Trust and Trusting Relationships

Most studies highlighted the importance of relational aspects and (quality) interactions in facilitating W/PCC [21, 38, 39, 40, 42, 44, 45, 46, 48, 49, 51, 52, 53, 54, 55, 57, 58, 60], with several emphasising the role of trust [21, 38, 43, 60]. One study found that professionals viewed reciprocal trust as central to ‘patient’‐centred maternity care’ (p. 3) [38]. Others noted that lack of emotional connection, insufficient knowledge of women's needs, and unclear boundaries hindered WCC [21, 44]. ‘*Trustful, safe communication’* (p. 389) between the woman and professionals was identified as essential to person‐centred maternity care [60], while another study linked poor midwife–woman relationships to reduced participation in care [53]. The consistency across studies suggests trust and relational quality are integral to effective W/PCC.

#### 4.3.3 Continuity of Care and/or Carer

Continuity of care and carer is closely linked to the relational aspects of care discussed. This review found that women consistently valued continuity as a key component of W/PCC [40, 48, 49, 55]. Where absent, it was viewed negatively [55]; women reported having to repeat their stories, encountering misinformation or experiencing poor handovers [55]. In Japan, positive perceptions of WCC were tied to respectful communication and midwifery continuity [49], while in the United States, Black birthing women associated continuity of care with higher PCC scores [40]. In Ireland, both women and professionals identified fragmented services and lack of continuity as barriers to WCC, concluding that continuity of carer models best support delivery of WCC [48]. However, UK midwives in one study questioned whether assigning a named midwife would automatically improve care quality [56], suggesting a potential disconnect between professionals and service‐users perspectives that warrants further exploration.

#### 4.3.4 The Psychosocial Parameters of W/PCC

Another aspect which has not reached consensus are the parameters of the psychosocial dimensions of W/PCC [21, 38, 52, 58]. While all stakeholders broadly acknowledge the importance of psychosocial factors in W/PCC, there remains ambiguity around the specific responsibilities involved in addressing the woman's psychosocial needs. One study found that maternity healthcare professionals felt obligated to address social barriers that might hinder ‘vulnerable*’ women from engaging with care (note: Authors use the Commonwealth Fund definition* as those without health insurance, from low‐income backgrounds or belonging to racial and ethnic minorities) [38]. A concept analysis further emphasised that WCC should not prioritise safety over the woman's experience; instead, physical and psychosocial aspects should be valued equally [21]. Another study, which assessed the most appropriate definition of PCC for maternity care, concluded that both social and clinical contexts must be considered [58]. Women too acknowledged the connection between a positive childbirth experience, which was strongly linked to elements of WCC, and psychosocial well‐being in the woman‐centred definition; which notes ‘[a positive birth experience] *can make women feel joy, confident, and/or accomplished and may have short‐and/or long‐term positive impacts on a woman's psychosocial well‐being’* [52].

#### 4.3.5 Level of Family Involvement

The involvement of family and friends is recognised as an important element, though studies varied in the extent to which this should occur [21, 38, 55, 58, 60]. An integrative review reported that quality interactions between the ‘patient’, family members and professionals were important [60]. Similarly, [58] the inclusion of ‘strong family involvement’ was a rationale for selecting Shaller's definition of PCC [61] as appropriate for maternity care. Adam's et al. study found that professionals viewed PCC as encompassing both women's preferences and family involvement, if this aligned with the woman's wishes. However, this was also influenced by whether professionals perceived the family's role as supportive or disruptive [38]. Fontein‐Kuipers et al.'s concept analysis noted the lack of reference to the infant and woman's partner in WCC, suggesting that the term ‘*family‐centred care’* may better reflect this dimension [21]. From women's perspectives, it was reported that participating women in Petit‐Steeghs et al.'s study valued the involvement of friends and family a component of their ‘interaction’ with maternity services (p. 80) [55].

### 4.4 WCC Within the Wider Maternity System

#### 4.4.1 Top‐Down Policy Directives—Understanding of W/PCC

Ambiguity also emerged in the only study to explore ‘top down’ maternity care policies. Dahlen et al. [59] examined policy documents from several HICs, including Australia, Canada, Ireland, New Zealand and the United Kingdom, to assess how the principles of WCC are interpreted and implemented. They found significant inconsistencies in decision‐making and autonomy, with no clear consensus on whether final decisions should rest with the woman or maternity healthcare professionals. Some policies (e.g., Australia and Northern Ireland) contained contradictory messages about autonomy, while others (e.g., Ireland, Scotland, Wales and Canada) emphasised sharing decision‐making among women, families and their healthcare providers. The study highlighted a lack of clarity at policy level regarding WCC and its implementation. Dahlen et al.'s study also noted that countries with strong midwifery professions were more likely to prioritise WCC, with emphasis on offering midwifery‐led models and diverse birth options [59].

#### 4.4.2 Impact of Policies and Protocols on WCC—Experiences of W/PCC

Policies, protocols and regulations that fail to consider their impact on WCC can hinder its provision [44, 57]. During COVID‐19, midwives reported that policies perceived as inconsistent with evidence or best practice created confusion in clinical settings [57]. For instance, reduced appointment times and limited contact impaired rapport‐building, while restrictions on birthing options constrained midwives' ability to provide WCC [57]. While these were remarkable times, the findings illustrate how conflicting policy can impede WCC. A 2015 Dutch study also found that the absence of ethical guidance undermined midwives' confidence in delivering WCC, concluding that clear definitions and guidance are essential [44] Yet, even with explicit strategic objectives, challenges in implementing WCC persist [48, 56]. In the United Kingdom, despite clear policy directives, significant variation in practice was reported [56]. In Ireland, WCC as study participants understood it was not routinely delivered despite policy commitment [48].

Relatedly, two studies suggest that midwives may exercise autonomy to circumvent policies or protocols they perceive as conflicting with WCC [47, 57]. Hollins Martin and Bull's UK study found that midwives often struggled to balance protocols with WCC, especially when medical procedures were perceived as unnecessary or potentially harmful [47]. To navigate this, they used strategies like advocating for women's choices, selectively presenting information, avoiding confrontation, and subtly influencing decisions to decline certain interventions [47]. Similarly, the study during COVID‐19 found that some midwives bypassed policies they felt misaligned with WCC by ‘*bending rules and pushing boundaries*’. (p. 480) [57].

#### 4.4.3 Organisational Structure of Maternity Care Services—Experiences of W/PCC

Organisational structures were found to influence and shape how care is delivered [39, 43, 44, 48, 53, 54, 57]. Studies from Ireland [48] and Switzerland [43] found that while professionals recognise the benefits of WCC, its implementation is hindered by institutional barriers, including organisational structures, staff competency and fragmented services [43, 48]. Several papers reviewed identified lack of time, heavy workload or work‐related stress as challenges for professionals in providing WCC [39, 43, 44, 48, 53, 54, 57]. The importance of organisational support in terms of structural, managerial and logistical factors which enable maternity care professionals to consistently implement WCC were emphasised [54, 60]; some additional considerations were in respect of knowledge, leadership, supervision, mentorship and resources [60].

#### 4.4.4 Culture of the Organisation on Care Provision—Experiences of W/PCC

The impact of organisational culture on care provision was evident. Studies reviewed suggest that the prevailing culture of the organisation and the attitudes of professionals working within the organisation impact on the (non)provision of WCC. Multiple papers explicitly noted that the prevailing medical model constrained professionals' in providing W/PCC [39, 48, 53, 54, 55, 56], with specific mention of this on labour wards [48]. Tensions were reported in relation to working cultures and professionals' perceptions about WCC [43, 44]. Conversely, it was found that shared beliefs about WCC generated support and connection through a mutual understanding [44]. Implementing a dedicated WCC model was found to enhance collaboration and clarify professional roles [53].

#### 4.4.5 WCC In Different Settings—Experiences of W/PCC

Certain approaches or philosophies of care were more strongly associated with the provision of W/PCC in maternity services [40, 42, 46, 49, 50, 55]. Two Dutch studies reported that the range of maternity care options available was perceived to support high levels of W/PCC, with community‐based midwives providing more W/PCC than hospital‐based midwives or obstetricians [46, 51]. Similarly, women who birthed at birth centres rated WCC highly and were more satisfied compared to clinics and hospitals [49]. This may reflect a discrepancy between the task‐oriented medical model of care and women's/clients' desire for a woman‐centred approach [55]. However, these findings were not consistent. In the United States, Black women and birthing people who received the majority of prenatal care from a professional who was not an obstetrician, such as a midwife or family practice physician, reported lower scores for PCC than those cared for by obstetricians [40]. Likewise, 48 migrant Hispanic women in the UnitedStates valued medical expertise and wanted doctors to lead decision‐making during pregnancy and birth [42].

## 5 Discussion

This rapid review set out to explore how W/PCC is understood and experienced within maternity services in HICs from the perspectives of stakeholders: women/service users and maternity healthcare professionals. Existing literature reveals persistent ambiguity and divergence in how W/PCC is defined and enacted [1, 4], with growing concerns that these models are often implemented tokenistically [7]. Despite W/PCC being a central tenet of maternity policy in many HICs [1, 62], a critical gap remains in understanding how these ideals are interpreted and operationalised in practice. This review directly addresses that gap through a focused synthesis which highlights the conceptual tensions and practical challenges which shape maternity care delivery in high‐resource settings. In doing so, it contributes a nuanced analysis of the disjuncture between policy aspirations and everyday clinical realities, an aspect often overlooked in prior reviews.

This review identified inconsistencies across all levels of maternity service provision; from top‐down policy directives [59], to institutional practices and in varying interpretations of W/PCC care among and within stakeholder groups which influence stakeholder interactions [42, 48, 52, 55]. Several studies noted that professionals' differing understandings influenced both their attitudes towards women and their approach to care [38, 43, 44, 45, 53, 56], while women themselves held diverse expectations of W/PCC [40, 42, 55]. Notably, few studies explicitly addressed how women's values and beliefs are incorporated into care, despite recognition in the broader literature of diversity in childbirth expectations [23, 24, 25, 26]. Therefore, it is plausible that some philosophies of care may not align, or could be in opposition to, women's aspirations for their birth. In Fontein‐Kuipers et al.'s study, midwives perceived that women who were assertive in their needs were both more likely to receive WCC, but somewhat paradoxically potentially viewed as demanding (p. 23) [44]. While this appears at odds with the ethos of W/PCC, it may reflect the pressures faced by professionals and resource constraints. Overall, the findings of this review underscore the need for deeper exploration of stakeholders' understandings and interactions across the childbirth continuum, with attention to the contextual dynamics within individual maternity systems.

In the studies reviewed, continuity of care and/or carer was acknowledged by many as an important aspect of WCC [21, 38, 48, 49, 53, 54, 60], often linked to building trust and fostering relationships, which were emphasised as central to W/PCC [21, 38, 43, 60]. Indeed, most studies reviewed highlighted the importance of interactions in care provision [21, 38, 39, 40, 42, 44, 45, 46, 48, 49, 51, 52, 53, 54, 55, 57, 58, 60]. However, continuity of care and/or carer as a domain of WCC did not reach consensus in studies reviewed, or in the wider literature: Corroborating Brady et al.'s suggestion [8, 9], some midwives did not view continuity of care and/or carer as being necessary to facilitating W/PCC [56]. In contrast, multiple studies affirmed Leap's definition, which prioritises continuity from a known caregiver [5], with women consistently valuing its presence and perceiving its absence as a shortfall [40, 48, 49, 55]. This inconsistency between professionals' and women's perceptions warrants further investigation. As Santana et al. argue [4], PCC must reflect the person's perspective; thus women's views should guide care priorities. Although Brady et al. conclude that continuity of care and/or carer is not strictly necessary for W/PCC, they nonetheless advocate for its implementation in all care settings [9].

Family involvement in W/PCC remains an area of ambiguity. This review [21, 38, 55, 58, 60] and the wider literature [1, 4] converge on the family's supportive role in maternity care, yet the extent and boundaries of their involvement are inconsistently defined. Fontein‐Kuipers et al. proposed renaming W/PCC to ‘*family‐centred care*’ to reflect inclusion of the infant and family [21]. However, another study noted that family involvement can be both beneficial and detrimental, depending on context and professional judgement [38]. Caution is warranted lest broadening the term has the paradoxical effect of overriding the birthing woman's autonomy. For example, in situations of domestic violence and abuse it could be dangerous to concede any degree of, or greater, control to the abuser [63] or other coercively controlling family members. Inclusion of the infant must also be considered carefully; an extreme example can be observed in Ireland's history where women's rights and autonomy were negated when a Constitutional amendment gave the unborn foetus equal status to that of the mother. This was shown to have a negative impact on care decisions and choice, with occasionally fatal consequences [64, 65]. As Leap's definition prescribes, the needs of the woman and her family are to be defined and negotiated by the woman *herself* [5].

This review highlights that some groups of women hold contrasting expectations and priorities about W/PCC. Studies included revealed that women of younger age, with lower socio‐economic status, minority ethnic backgrounds, unmarried or non‐heterosexual groups reported lower PCC scores [40, 41]. These groups placed more value on friendliness and warm interactions, without emphasising choice, control or leading care decisions [42, 55]. In contrast, women with higher levels of health literacy and/or better health status reported higher PCC scores [40, 41], suggesting they were more likely to expect and engage in participatory care. As W/PCC should be accessible for all women, further investigation into the causes of these disparities and strategies to address them is merited.

In recent years, an increasing number of women are choosing to birth outside the maternity system in a practice known as ‘freebirth’ [66, 67]. Defined as the intentional decision to birth without a regulated/registered healthcare professional, ‘freebirth’ is rarely a woman's first choice. A recent systematic review found it often follows restricted access to choice or previous negative birth experiences [67]. If ‘freebirth’ is used as barometer of women's attitude to maternity services, then deeper exploration of care provision must be undertaken to ensure that birthing women's needs are being met within maternity services. Furthermore, greater focus needs to be placed on facilitating W/PCC for women with complex pregnancies. WCC should not be reserved for those with uncomplicated pregnancies [68], rather the increased likelihood of interventions should heighten the need for shared decision‐making which is respectful of the woman's values, beliefs and lifestyle.

The ambiguity illustrated above is also reflected at strategy level: The only study which compared policy documents from HICs with comparable maternity services (Australia, Canada, Ireland, New Zealand and United Kingdom) revealed variation in the parameters and operationalisation of woman‐centred maternity care in the nine policy documents reviewed. It found that there were conflicting messages observed within individual strategies [59]. Such ambiguity from top‐down policy directives could suggest a level of ‘tokenism’, or lack of understanding, about facilitating WCC. Furthermore, it found that in Australia and Ireland, evidence‐based recommendations and consumer consultations were overridden or diluted in favour of the prevailing medical approaches to birth [59]. Decisions such as these invalidate any intention to provide W/PCC.

Relatedly, another prominent theme emerging from this review is the importance of genuine organisational commitment to WCC. Without adequate scope for the provision WCC, it negates any policy intention. Multiple studies reported that time constraints, workplace stress and workload pressures limited professionals' capacity to provide WCC [39, 43, 44, 48, 53, 54, 57]. The need for clearer implementation strategies and practice guidelines were emphasised in several studies reviewed [21, 43, 44, 53, 56, 58]. Where WCC interventions were trialled [39, 53, 54], clarity around professionals' roles, practice guidance and justification for providing more WCC were perceived positively. This review underscores that if genuine WCC is to be facilitated, it needs to be defined unambiguously in the context of each service or system, operationalised across all professional stakeholder groups who interact with women in maternity services, and embedded in all institutional protocols and procedures. Accordingly, Stulz et al.'s study further recommended that policymakers and institutions consider how policy decisions impact on midwives' (and all maternity healthcare professionals') ability to provide quality WCC [57].

The wider literature acknowledges that organisational culture may affect the quality of care from within healthcare and maternity care settings [4, 69, 70, 71]. Santana et al. explicitly emphasise the importance of workplace culture in their conceptual framework on how to practice PCC [4]. Depending on the prevailing ethos, the culture of the setting can enable or hinder facilitating W/PCC. One reviewed study reported that a culture which openly values and applies the principles of WCC was more likely to have expectations or accountability about its provision [44]. Conversely, in settings where WCC was not the normative approach, professionals did not notice any obligation from colleagues for facilitating it [39, 48, 53, 54, 55, 56]. These findings support Santana et al.'s assertion [4] that organisational/institutional ‘buy‐in’ is imperative when aiming to provide genuine P/WCC. In maternity care, this includes ensuring time and space for relationship‐building and supporting physiological birth processes, when clinically appropriate. Without enabling professionals to engage fully with birthing women, WCC risks becoming superficial. It is apparent from the papers reviewed that, if the medical model approach prevails or is the normative practice, then a genuine commitment to changing the culture of the organisation must be instigated. Two studies reviewed mentioned the importance of providing opportunities for reflective practice in this regard [39, 53].

International literature [72, 73, 74, 75, 76, 77] has shown that care provided by midwives yields better outcomes for women with low risk of obstetric complications compared to standard obstetric care, largely due to the relationship fostered through continuity of care and/or carer [77, 78, 79]. Several studies in this review found that WCC was perceived to be more abundant in midwifery‐led care settings [46, 49, 50, 51], ostensibly due to the greater continuity of care and/or carer inherent in such models. Notably, this review identified that even within midwifery‐led settings, smaller sized practices with fewer midwives received significantly higher scores [51], suggesting that fewer care providers may facilitate stronger relationships. Additionally, in the broader literature, hospitals' policies and procedures were often found to be impediments to this type of relational care [73, 77, 80]. However, of note and demonstrating the validity of exploring countries with comparable economic profiles and maternity care systems, two studies reviewed explicitly noted that overall care was perceived to be of a high standard, with high levels of WCC from all care settings [46, 51].

## 6 Conclusion

This rapid review identified considerable variation and inconsistency in both the interpretation and implementation of W/PCC across all levels of maternity services. These discrepancies were evident from overarching policy directives to the perceptions and expectations of individual stakeholders involved in maternity care (e.g., women and maternity care professionals). Complexity theory, which views healthcare systems as complex adaptive systems, offers a useful lens for understanding these inconsistencies. Within such systems, diverse agents (e.g., service‐users, professionals and institutions) interact in non‐linear, unpredictable ways, producing emergent behaviours that challenge top‐down control and require responsive, context‐sensitive approaches [81, 82]. Concepts such as emergence, self‐organisation [83] and local rationalities [84] could help explain how decisions that deviate from W/PCC principles may still make sense to those involved. Conversely, this review found that some professionals may circumvent protocols they believe are misaligned with best practice [47, 57]. This is important because while the practice of bending rules to facilitate WCC might be beneficial to one birthing woman, it does little to address the existing systemic, policy or protocol issues that may be restricting the provision of WCC to all women. These findings support the need for further research using complexity‐informed approaches to examine how W/PCC is operationalised within specific maternity systems. Understanding what facilitates or hinders WCC in context is essential for developing meaningful, locally relevant recommendations.

## 7 Strengths and Limitations

A key strength of this rapid review is its focused scope, offering a fresh perspective on WCC in maternity services. Inclusion of diverse maternity healthcare professionals also broadened the insights beyond midwifery. However, limitations include the exclusion of non‐English language studies, which may have omitted relevant research. While only 10% of studies underwent double review due to resource constraints, there is evidence to suggest that once agreement between reviewers is greater than 80% this approach is considered sufficiently reliable [29]. Despite these limitations, the findings are useful to the development of woman‐centred maternity services in comparable healthcare contexts.

## Author Contributions

Conceptualisation: S.G. Methodology: S.G., L.C., D.D. and G.S. Formal analysis: S.G. Investigation: S.G., L.C., D.D. and G.S. Data curation: S.G., L.C. and D.D. Writing – original draft: S.G. Writing – review and editing: L.C., D.D. and G.S. Supervision: L.C. and D.D. Project administration: S.G.

## Ethics Statement

This rapid review did not involve the collection of primary data from human participants. Therefore, ethical approval was not required. The review was conducted in accordance with the principles of research integrity and transparency. All sources included in the review were publicly available and appropriately cited. No personal or sensitive data were accessed or used in this study.

## Conflicts of Interest

The authors declare no conflicts of interest.

## Supporting information


**Data S1.** Supporting Information.

## Data Availability

Data sharing is not applicable to this article as no new data were created or analyzed in this study.
