# Supplementary material for: How Woman‐Centred Care Is Experienced and Understood in Maternity Services by Women and Professionals: A Rapid Review
Source: Scand J Caring Sci. 2025 Jul 10;39(3):e70086. doi: 10.1111/scs.70086 (PMC12243450; doi:10.1111/scs.70086)
Supplement: Supplementary file 1 — Data S1. Supporting Information. [file SCS-39-0-s001.docx]

**Supplementary Information File 1**

**Table 1: A comparison of three definitions of woman-centred care in maternity services**

|  | **Brady et al (2024)**  **(1)** | **Fontein-Kuipers et al (2018)**  **(2)** | **Leap (2009)**  **(3)** |
| --- | --- | --- | --- |
| **Aim/purpose** | To construct a standardised, internationally informed definition of woman-centred care. | To refine and advance the concept of woman-centred care in midwifery research and practice. | To debate the conceptual implications of woman-centred care and its political and feminist underpinnings. |
| **Methodology** | Evolutionary Concept Analysis. | Advanced Concept Analysis using principal-based evaluation. | Discussion based analysis using historical, political and linguistic analysis to critique how woman-centred care is conceptualised and applied in practice. |
| **Interdisciplinary approach** | International maternity care professionals; including midwives, nurses, educators and policy makers. | Midwifery professionals only. | Focus is midwifery, but situated within wider maternity systems. |
| **Outcome** | Constructed a formal evidence-based definition for use in policy, education and clinical practice. | Provides a conceptual refinement of woman-centred care, but not a standardised definition. | A broad definition is provided. Challenges how woman-centred care is interpreted, used, and influenced by power structures. It argues for a woman-focused feminist approach. |
| **Key attributes** | Choice and control.  Empowerment.  Midwife woman relationship. | Relational focus between midwife and woman.  Equity and shifting control in care.  Trust, reciprocity and autonomy; of woman and midwife. | Shifting power away from institutions and towards women.  Individualised care. |
| **Choice & control** | Women must have full control over care decisions, with midwife acting as facilitator, not a gatekeeper. | Midwife facilitates informed choice, ensure equity in decision-making. | Woman-centred care is about giving power to women, ensuring they dictate care on their terms. |
| **Continuity of Care** | Not a core attribute of woman-centred care, continuity is beneficial but not required for care to be woman-centred. | Seen as important but not essential. Focus is on relational continuity rather than systems-based models. | Essential for woman-centred care. Having a known midwife throughout pregnancy and birth is fundamental. |
| **Autonomy & decision-making** | Women should be fully autonomous in decision-making, supported by evidence-based care and shared decision-making. | Autonomy is important but it is balanced with midwifery expertise; decisions should be made in partnership. | The woman is the primary decision-maker. The role of the midwife is to ensure her empowerment and control. |
| **Definition** | *Woman-centred care ensures the woman has choice and control in her childbearing experience. Relationships built on reciprocal trust and respect foster empowerment, which recognises the woman's innate ability to meet her own needs. This is upheld through evidence-based shared and informed decision-making that supports the woman to navigate complex health systems and ultimately improves health outcomes for both the woman and neonate.* | *Woman-centred care is a philosophy and a consciously chosen tool for the care management of the childbearing woman, where the collaborative relationship between the woman - as an individual human being - and the midwife - as an individual and professional - is shaped through co-humanity and interaction; recognising and respecting one another’s respective fields of expertise. Woman-centred care has a dual and equal focus on the woman’s individual experience, meaning and manageability of childbearing and childbirth, as well as on health and wellbeing of mother and child. Woman-centred care has a reciprocal character but fluctuates in equality and locus of control.* | *Woman-centred care is a concept. It implies that: midwifery:*  *Focuses on the woman’s individual needs, aspirations and expectations, rather than the needs of the institution or professionals.*  *Recognises the need for women to have choice, control and continuity from a* ***known*** *caregiver or caregivers*  *Encompasses the needs of the baby, the woman’s family and other people important to the woman, as defined and negotiated by the woman herself.*  *Follows the woman across the interface of community and acute settings.* |

**References and bibliography**

1. Brady S, Gibbons KS, Bogossian F. Defining woman-centred care: A concept analysis. Midwifery. 2024;131:103954.

2. Fontein-Kuipers Y, De Groot R, Van Staa A. Woman-centered care 2.0: Bringing the concept into focus. European journal of Midwifery. 2018;2.

3. Leap N. Woman-centred or women-centred care: does it matter? British Journal of Midwifery. 2009;17(1):12-6.
